# Asymmetric Dissociative Tunneling Ionization of Tetrafluoromethane in *ω* − 2*ω* Intense Laser Fields

**DOI:** 10.3389/fchem.2022.857863

**Published:** 2022-04-14

**Authors:** Hiroka Hasegawa, Tiffany Walmsley, Akitaka Matsuda, Toru Morishita, Lars Bojer Madsen, Frank Jensen, Oleg I. Tolstikhin, Akiyoshi Hishikawa

**Affiliations:** ^1^ Graduate School of Science, Nagoya University, Nagoya, Japan; ^2^ School of Chemistry, University of Edinburgh, Edinburgh, United Kingdom; ^3^ Institute for Advanced Science, The University of Electro-Communications, Chofu-shi, Tokyo, Japan; ^4^ Department of Physics and Astronomy, Aarhus University, Aarhus, Denmark; ^5^ Department of Chemistry, Aarhus University, Aarhus, Denmark; ^6^ Moscow Institute of Physics and Technology, Dolgoprudny, Russia; ^7^ Research Center for Materials Science, Nagoya University, Nagoya, Japan

**Keywords:** coherent control, intense laser fields, tunneling ionization, molecular dissociation, tetrafluoromethane

## Abstract

Dissociative ionization of tetrafluoromethane (CF_4_) in linearly polarized *ω*-2*ω* ultrashort intense laser fields (1.4 × 10^14^ W/cm^2^, 800 and 400 nm) has been investigated by three-dimensional momentum ion imaging. The spatial distribution of ${CF}_{3}^{+}$ produced by CF_4_ → ${CF}_{3}^{+}$ + F + e^−^ exhibited a clear asymmetry with respect to the laser polarization direction. The degree of the asymmetry varies by the relative phase of the *ω* and 2*ω* laser fields, showing that 1) the breaking of the four equivalent C-F bonds can be manipulated by the laser pulse shape and 2) the C-F bond directed along the larger amplitude side of the *ω*-2*ω* electric fields tends to be broken. Weak-field asymptotic theory (WFAT) shows that the tunneling ionization from the 4*t*_2_ second highest-occupied molecular orbital (HOMO-1) surpasses that from the 1*t*_1_ HOMO. This predicts the enhancement of the tunneling ionization with electric fields pointing from F to C, in the direction opposite to that observed for the asymmetric fragment ejection. Possible mechanisms involved in the asymmetric dissociative ionization, such as post-ionization interactions, are discussed.

## 1 Introduction

Shaped intense laser fields with a field intensity of ∼ 10^14^ W/cm^2^ have attracted considerable attention in the last decades for their capability to manipulate ultrafast electronic and nuclear dynamics of atoms, molecules, and solids. Armed with the electric field exerting a force on the electrons comparable to that of the Coulomb potential in a molecule, shaped laser pulses enable us to drive electrons in a nonperturbative manner to exploit unique properties from the targets. The application has been demonstrated in controls of high-order harmonic generation (Bartels et al., 2000; Pfeifer et al., 2005; Winterfeldt et al., 2008), photoemission (Bardeen et al., 1997; Wollenhaupt and Baumert, 2011; Eickhoff et al., 2021), and chemical reactions (Levis et al., 2001; Assion et al., 1998; Levis and Rabitz, 2002; Hishikawa et al., 2020).

**FIGURE 1:**
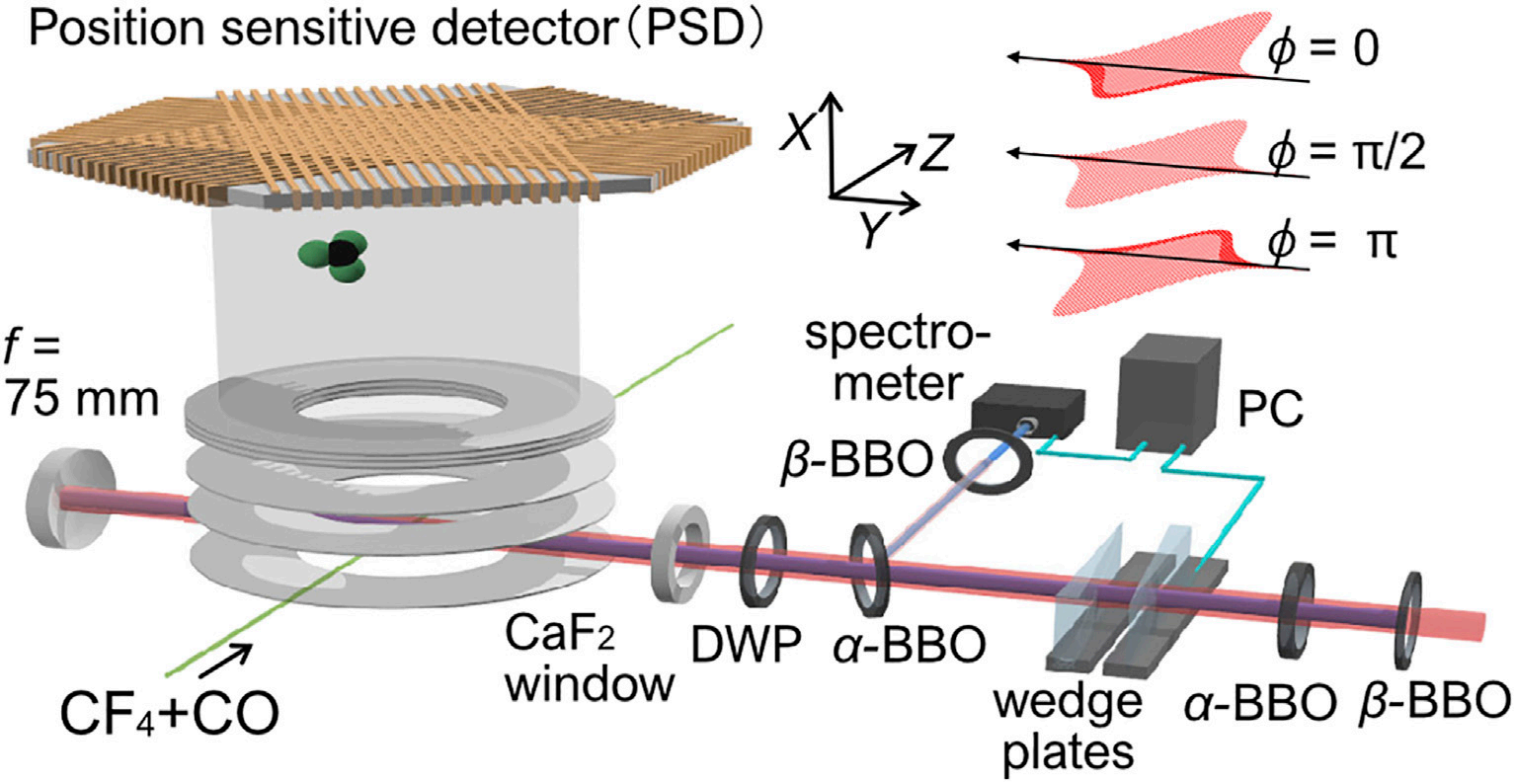
Schematic of the experimental setup. The output from a Ti: Sapphire regenerative laser amplifier system (800 nm, 1 kHz, 50 fs) was introduced to a *β*-BBO crystal (type-I) to generate a second-harmonic pulse (400 nm). The time delay between the fundamental (*ω*) and the second harmonics (2*ω*) pulse was compensated by two birefringent *α*-BBO crystals. The two-color relative phase was stabilized by a pair of fused-silica wedge plates controlled by the active feedback locking to the 2*ω*-2*ω* interference spectrum. The polarization of the fundamental and the second harmonic pulse was set parallel by a dual-wavelength plate (DWP). For the phase calibration, CO gas is mixed with the sample gas of CF_4_.

Laser pulse shaping can be accomplished by a spatial amplitude and phase modulator placed on a Fourier transform plane in a 4*f* setup (Bardeen et al., 1997; Levis et al., 2001; Assion et al., 1998; Eickhoff et al., 2021). Alternatively, one can synthesize the laser waveform by coherent superposition of pulses with different colors (Chan et al., 2011; Manzoni et al., 2015), which has been used to control high harmonic generation (Takahashi et al., 2010; Neyra et al., 2018) and multiphoton and tunneling ionization of atoms and molecules (Eickhoff et al., 2021; Ohmura and Saito, 2020; Ohmura et al., 2021). Among others, the *ω*-2*ω* laser fields, consisting of the fundamental and the second harmonics, have been widely used for understanding the mechanisms of laser tunneling ionization and chemical reaction control in intense laser fields. In the case of linear polarization along the *Z* direction, the *ω*-2*ω* electric fields may be expressed as follows (Endo et al., 2019):

$$\begin{array}{ccc} & F\left(t\right)=F\left(t\right)e_{Z}, & \end{array}$$ (1)

$$\begin{array}{ccc} & F\left(t\right)={\bar{F}}_{\omega }\left(t\right)\cos\left(\omega t\right)+{\bar{F}}_{2\omega }\left(t\right)\cos\left(2\omega t+\phi \right), & \end{array}$$ (2)

where ${\bar{F}}_{\omega }\left(t\right)$ and ${\bar{F}}_{2\omega }\left(t\right)$ represent the envelopes of the fundamental and the second harmonic pulses, respectively, and *ϕ* is the two-color relative phase. The unit vector along the *Z*-axis is denoted as **e**_*Z*_. Typical *ω*-2*ω* electric fields are illustrated in Figure 1, showing that the direction and degree of asymmetry vary by phase *ϕ* for a given ratio of the *ω* and 2*ω* field intensities.

Asymmetric fragment ejection through directional bond-breaking has been observed for various molecules in the *ω*-2*ω* intense laser fields. For HD (Sheehy et al., 1995), NO (Endo et al., 2019; Li et al., 2011), CO (Li et al., 2011; Ohmura et al., 2011; Ohmura et al., 2014), OCS (Ohmura et al., 2014; Endo et al., 2022), and CH_3_X (X = F, Cl, Br, I) (Ohmura et al., 2006a; Ohmura et al., 2006b; Walt et al., 2015), the directional fragment ejection has been observed. The observed asymmetric distribution of fragment ions is interpreted as a result of orientation-selective tunneling ionization followed by dissociation in intense *ω*-2*ω* laser fields. Molecular tunneling ionization has been discussed intensively in the last decade, showing that many of the characteristic properties can be understood in terms of the shape of molecular orbitals (MOs) and their direction of electric dipole moments. Because of the asymmetric MOs and the non-zero dipole moments of the linear heteronuclear molecules mentioned above, tunneling ionization is enhanced in one direction along the molecular axis compared to the other, resulting in orientation-selective ionization.

The asymmetric fragment ejection is also observed with symmetric molecules such as D_2_ (Ray et al., 2009; Wanie et al., 2015), H_2_O (Kechaoglou et al., 2019), CO_2_ (Endo et al., 2016; Endo et al., 2017), and C_2_H_2_ (Song et al., 2015). For D_2_ (Ray et al., 2009; Wanie et al., 2015), electron localization is induced by the coherent superposition of two cationic states through interaction with two kinds of photons of the fundamental and second harmonic, resulting in asymmetric D^+^ ejection. For C_2_H_2_ (Song et al., 2015), H^+^ ejection associated with breaking the C-H bond shows clear asymmetry with respect to the laser polarization. The observed selectivity is suggested to be produced by laser-induced coupling of HOMO and HOMO-1, 2 states. For CO_2_ (Endo et al., 2016; Endo et al., 2017), asymmetric ejection of O^+^ was observed on the larger amplitude side of the *ω*-2*ω* laser fields. This is consistent with the results of a theoretical calculation of nuclear wave packet dynamics on the potential energy surfaces (PES) of ${CO}_{2}^{2+}$ in *ω*-2*ω* intense laser fields (Sato et al., 2003), demonstrating the chemical reaction control by laser manipulation of PES.

**FIGURE 2:**
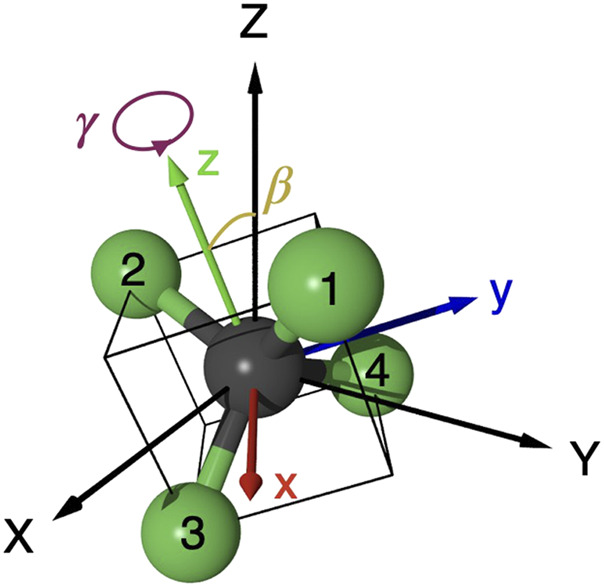
Molecular orientation of CF_4_ in the laboratory frame (*X*, *Y*, *Z*), where the polarization direction of the linearly polarized *ω*—2*ω* laser fields is directed along the *Z*-axis. The molecular principal axis (*C*_2_ axis) is along the *z*-axis of the molecular frame (*x*, *y*, *z*). The orientation is specified by the Euler angles (*α*, *β*, *γ*). Because of the axial symmetry around the electric field *F* one can set *α*=0 without losing generality.

**FIGURE 3:**
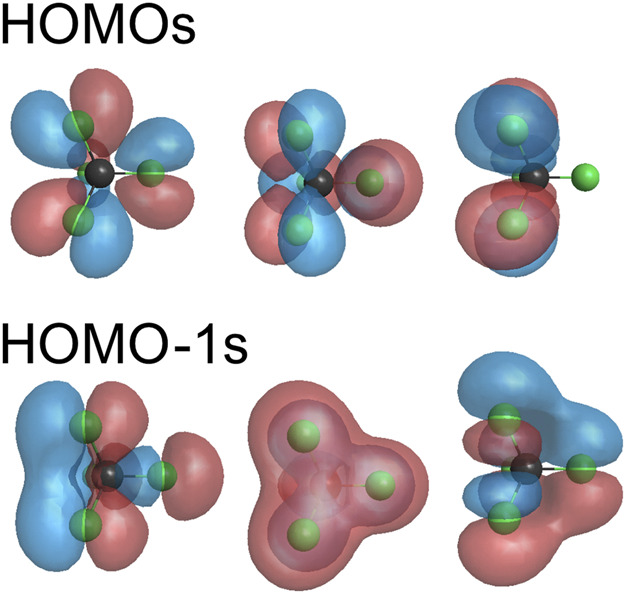
Highest-occupied molecular orbitals HOMO (1*t*_1_) and HOMO-1 (4*t*_2_). Both HOMO and HOMO-1 are triply degenerated.

This study discusses the feasibility of applying the *ω*-2*ω* reaction control to more complex symmetric molecules. More specifically, we study a tetrahedral molecule, tetrafluoromethane (CF_4_), which has four equivalent C-F bonds in the equilibrium structure in *T*_d_ symmetry (Figure 2) to see if directional ejection of the fragment can be induced by asymmetric laser fields. The electronic configuration is … ${\left(1e\right)}^{4}{\left(4t_{2}\right)}^{6}{\left(1t_{1}\right)}^{6}$ in the ground state. The highest-occupied MO (HOMO), 1*t*_1_, is triply degenerated (see Figure 3). We discuss dissociative ionization in *ω*-2*ω* intense laser fields:

$${CF}_{4}\to {CF}_{3}^{+}+F+e^{-}.$$ (3)

**FIGURE 4:**
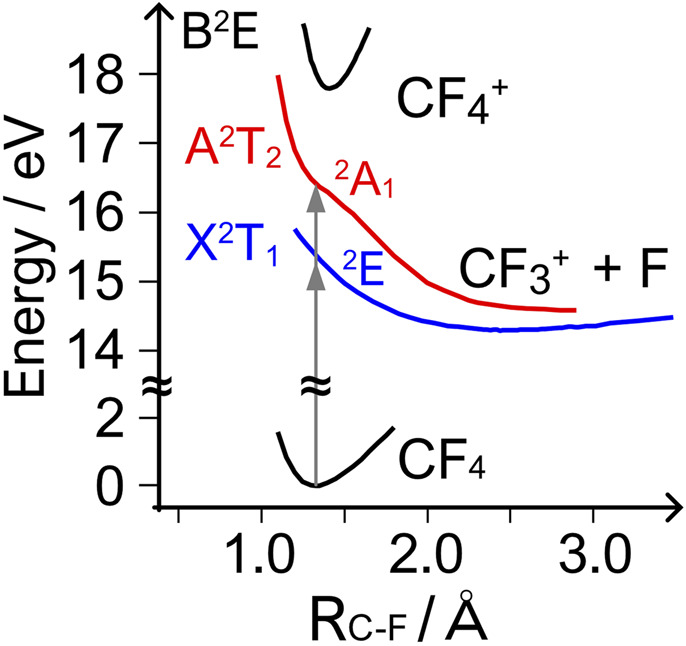
Schematic potential energy curves of selected electronic states of CF_4_ and ${CF}_{4}^{+}$ as a function of the internuclear distance *R*_C-F_ between F and C in the CF_3_ group [reproduced from Tang et al. (2013)].

The dissociative ionization has been subjected to single-photon (Brehm et al., 1974; Creasey et al., 1990; Hikosaka and Shigemasa, 2006; Tang et al., 2013; Larsen et al., 2018; Pertot et al., 2017) and electron impact (Hossen et al., 2018) studies. The process is characterized by the ultrashort lifetime ( < 40 fs) (Pertot et al., 2017) on the repulsive PESs leading to the ${CF}_{3}^{+}$ + F asymptote in both the ground and the first excited states of ${CF}_{4}^{+}$ as shown in Figure 4. The repulsive PESs imply that the CF_4_ can serve as a unique benchmark to elucidate how the tunneling ionization of polyatomic molecules proceeds in intense laser fields because fragments can be produced by direct dissociation without additional interaction with the laser fields (Fujise et al., 2022).

The paper is organized as follows. We first describe the experimental setup for the three-dimensional momentum imaging of ${CF}_{3}^{+}$ fragment ions produced by dissociative ionization in Eq. 3 in linearly polarized *ω*-2*ω* intense laser fields (50 fs, 1.4 × 10^14^ W/cm^2^, 800 and 400 nm). Then, we present the experimental results on the asymmetry in the ejection of ${CF}_{3}^{+}$ and its dependence on the relative phase *ϕ* between the *ω* and 2*ω* laser fields. Finally, the obtained results are compared with theoretical predictions by the weak-field asymptotic theory (WFAT) (Tolstikhin et al., 2011) for tunneling ionization.

## 2 Experiment

The schematic of the experimental setup is shown in Figure 1. Details have been described previously (Endo et al., 2019). Briefly, the output from a Ti: Sapphire regenerative laser amplifier system (800 nm, 1 kHz, 50 fs) was introduced to an inline *ω*-2*ω* pulse generator. After generation of the second-order harmonics (400 nm, ∼80 fs) by a type-I *β*-BBO crystal, the time delay between the *ω* and 2*ω* pulses was compensated by two birefringent *α*-BBO crystals. The relative phase between the two-color was controlled by a pair of fused silica wedge plates. The relative phase of the two-color laser pulses was stabilized by active feedback control of the wedge plate utilizing the 2*ω*-2*ω* interference spectrum. The polarization direction of the fundamental and second harmonic pulses was set parallel with each other by a true zero-order dual-wavelength plate and introduced into an ultrahigh vacuum chamber. The *ω*-2*ω* laser pulse was focused onto a diffusive molecular beam by a focusing mirror (*f* = 75 mm). Fragment ions generated by the interaction with *ω*-2*ω* intense laser fields were guided to a delay-line anode position-sensitive detector (PSD) by a static electric field. The three-dimensional momentum (*p*_*X*_, *p*_*Y*_, *p*_*Z*_) of each fragment ion was obtained from the arrival position (*Y*, *Z*) at the detector and the time of flight (*t*). The kinetic energy release (KER) was calculated from the momentum of ${CF}_{3}^{+}$ , $p_{{\text{CF}}_{3}^{+}}$ , where we assume the momentum conservation between ${CF}_{3}^{+}$ and the counterpart fragment F atom, **p**_F_ = – $p_{{\text{CF}}_{3}^{+}}$ . Under this approximation, the KER is expressed as

$$E_{\text{kin}}=\frac{m_{F}+m_{{\text{CF}}_{3}^{+}}}{2m_{F}m_{{\text{CF}}_{3}^{+}}}|p_{{\text{CF}}_{3}^{+}}{|}^{2},$$ (4)

where $m_{{\text{CF}}_{3}^{+}}$ and *m*_F_ are the masses of the ${CF}_{3}^{+}$ fragment ions and F atoms, respectively.

The intensities of the laser fields were estimated to be *I*_*ω*_ = 1.15 × 10^14^ W/cm^2^ and *I*_2*ω*_ = 2.6 × 10^13^ W/cm^2^, respectively. The total field intensity is *I*_*ω*+2*ω*_ = *I*_*ω*_ + *I*_2*ω*_ = 1.4 × 10^14^ W/cm^2^ with a ratio of *I*_2*ω*_/*I*_*ω*_ = 0.23. A mixture of CF_4_ and CO was used as the sample gas. The absolute phase difference *ϕ* between *ω* and 2*ω* pulses at the focal point was determined by the phase dependence of Coulomb explosion of CO, CO → C^+^ + O^+^ + 2e^−^, where C^+^ is ejected more to the smaller amplitude side of the *ω*-2*ω* electric fields than to the opposite (Li et al., 2011).

## 3 Results and Discussion

### 3.1 Fragment Momentum Distribution

**FIGURE 5:**
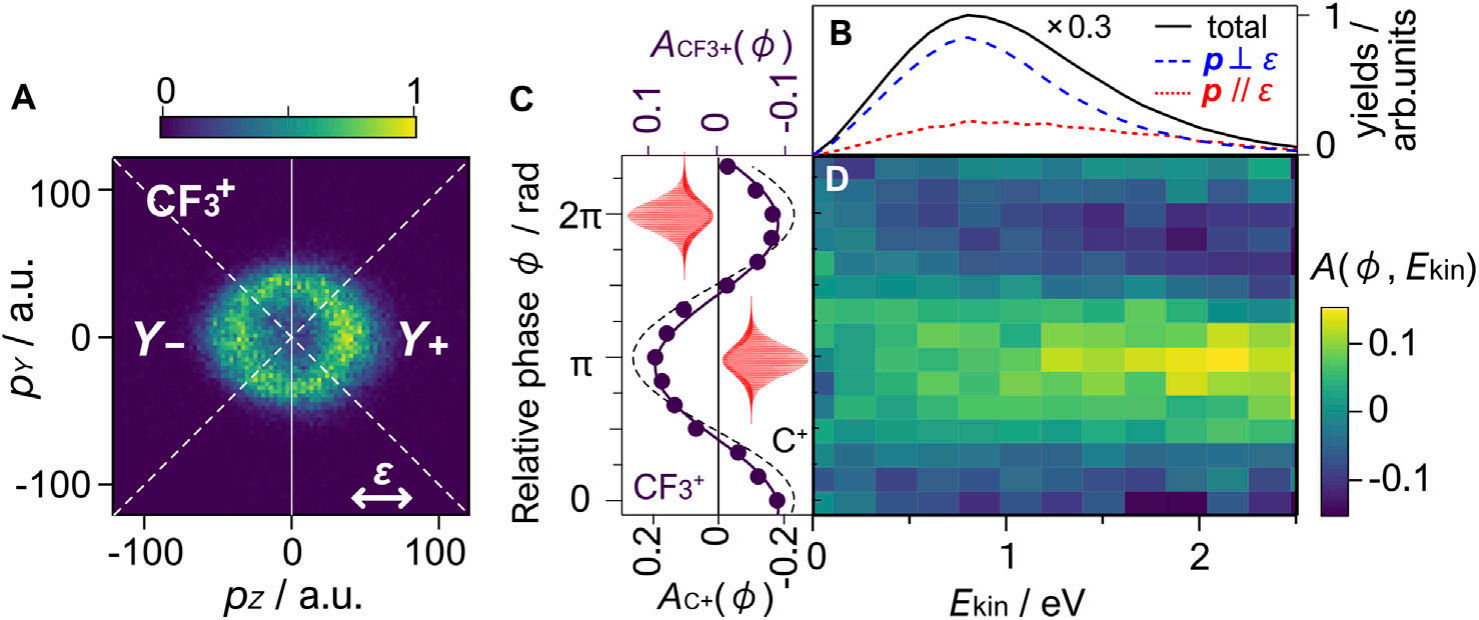
**(A)** Momentum image of ${CF}_{3}^{+}$ fragment ions produced in *ω*-2*ω* intense laser fields (50 fs, 1.4 × 10^14^ W/cm^2^, 800 and 400 nm), averaged over the relative phase (0 ≤ *ϕ* ≤ 2*π*). The image is a slice of the three-dimensional ion momentum distribution in the *Y*-*Z* plane with a thickness of |*p*_*X*_| < 10 a.u. The arrow represents the direction of the laser polarization. **(B)** Total kinetic energy release spectra of ${CF}_{4}^{+}\to {CF}_{3}^{+}$ + F (solid) plotted together with the parallel (dotted) and perpendicular (dashed) components, defined by the polar angles of 0° ≤ *θ* ≤ 20° and 75° ≤ *θ* ≤ 90°, respectively. The total spectrum is multiplied by 0.3. **(C)** The asymmetry parameter $A_{{\text{CF}}_{3}^{+}}\left(\phi \right)$ for the ${CF}_{3}^{+}$ fragment ions with an acceptance angle of 45°(solid circle) and the results of the least-square fitting (solid line) (see text). The fitting results for the C^+^ ion produced by the Coulomb explosion of CO (4 eV $\leq E_{\text{kin}}\leq$ 8 eV) are also plotted (dashed line). The laser pulse shapes at *ϕ* = 0 and *π* are shown. **(D)** Two-dimensional plot of the asymmetry parameter *A*(*ϕ*, *E*_kin_) for ${CF}_{3}^{+}$ .

Figure 5A shows the momentum image of ${CF}_{3}^{+}$ that dominates the time-of-flight spectrum, reflecting the repulsive nature of the PES of ${CF}_{4}^{+}$ . The KER spectrum shows a broad single peak at *E*_kin_ = 0.8 eV as observed in the previous studies (Tang et al., 2013; Larsen et al., 2018; Hossen et al., 2018; Hikosaka and Shigemasa, 2006; Fujise et al., 2022). The ${CF}_{3}^{+}$ momentum image in Figure 5A shows an elliptic distribution. The peak momentum values along the *Z*-axis and *Y*-axis are 35 a.u. and 30 a.u., respectively, showing that ${CF}_{3}^{+}$ is emitted with a larger momentum along the laser polarization direction. Figure 5B shows the KER spectra obtained for parallel (0° ≤ *θ* ≤ 20°) and perpendicular (75° ≤ *θ* ≤ 90°) components to the laser polarization direction, where *θ* is the polar angle from the *Z*-axis. The parallel component shows a broader peak at 0.9 eV extending to a higher KER region than the perpendicular component. The perpendicular component has a sharper peak at 0.8 eV, indicating that different pathways contribute to the dissociative ionization. The previous electron impact study at an energy of 67 eV (Hossen et al., 2018) shows that contributions from five different molecular orbitals 1*t*_1_, 4*t*_2_, 1*e*, 3*t*_2_, and 4*a*_1_ overlap within the peak. The KER spectrum associated with the ionization from HOMO (1*t*_1_) exhibits a peak at ∼0.9 eV, while a broader peak appears at a slightly higher energy region for HOMO-1 (4*t*_2_). This suggests that both the X^2^T_1_ ground state and A^2^T_2_ first excited state contribute to the dissociative ionization in the *ω*-2*ω* intense laser fields, although it is difficult to estimate the relative contributions from these orbitals by the present experimental results. It is worth noting that the dissociation from the 1*e* HOMO-2 state may also contribute to the KER spectrum (Larsen et al., 2018) through internal conversion from the B^2^E to the A^2^T_2_ state (Maier and Thommen, 1980).

### 3.2 Asymmetric Dissociative Ionization of CF_4_

To understand how CF_4_ responds to different shapes of the laser pulse, we focus on the spatial asymmetry in the fragment distribution. For a quantitative discussion, the asymmetry parameter,

$$A\left(\phi \right)=\frac{Y_{+}\left(\phi \right)-Y_{-}\left(\phi \right)}{Y_{+}\left(\phi \right)+Y_{-}\left(\phi \right)},$$ (5)

is introduced, where *Y*_+_ and *Y*_−_ represent the yields of ions with positive and negative momenta within a 45° acceptance angle along the laser polarization direction (*Z-*axis), respectively (see Figure 5A). Figure 5C plots the obtained asymmetry parameters for ${CF}_{3}^{+}$ , $A_{{\text{CF}}_{3}^{+}}$ (*ϕ*), together with those obtained for C^+^ produced from the Coulomb explosion of CO used for the phase calibration. The asymmetry parameter shows a 2*π* periodic dependence on the two-color relative phase. The least-squares fitting to *A*(*ϕ*) = *A*_0_cos(*ϕ*-*ϕ*_0_) provides *A*_0_ = 0.09(1) and *ϕ*_0_ = 0.9(1) *π*, where numbers in the parentheses represent uncertainties. The results show that ${CF}_{3}^{+}$ prefers being emitted on the smaller electric field side of the asymmetric laser fields. In other words, the dissociative tunneling ionization is enhanced when the lager amplitude side of the *ω*-2*ω* electric fields points from C to F. Figure 5D shows the KER-resolved asymmetry parameter,

$$A\left(\phi ,E_{\text{kin}}\right)=\frac{Y_{+}\left(\phi ,E_{\text{kin}}\right)-Y_{-}\left(\phi ,E_{\text{kin}}\right)}{Y_{+}\left(\phi ,E_{\text{kin}}\right)+Y_{-}\left(\phi ,E_{\text{kin}}\right)}.$$ (6)

An increase in the asymmetry amplitude to *A*_0_ ∼ 0.12 is observed in higher KER region where contributions from the A^2^T_2_ state of ${CF}_{4}^{+}$ is observed. The maximum and minimum of the asymmetry parameter are seen at *ϕ* ∼ *π* and 0, respectively, over the KER range investigated.

### 3.3 Comparison With Tunneling Ionization Theory

#### 3.3.1 Tunneling Ionization Rates

Theoretical calculations of the tunneling ionization rate of CF_4_ were carried out by WFAT (Tolstikhin et al., 2011). The tunneling ionization rate is expressed as (Madsen et al., 2012)

$$\Gamma \left(\beta ,\gamma \right)=|G_{00}\left(\beta ,\gamma \right){|}^{2}W_{00}\left(F\right).$$ (7)

The structure factor *G*_00_(*β*, *γ*) describes the dependence on the molecular orientation relative to the laser electric field *F* defined by the Euler angles (*α*, *β*, *γ*) (Zare, 1988). The field factor *W*_00_(*F*) is given as

$$W_{00}\left(F\right)=\frac{\varkappa }{2}{\left(\frac{4\varkappa^{2}}{F}\right)}^{2/\varkappa -1}\exp\left(-\frac{2\varkappa^{3}}{3F}\right),$$ (8)

which defines the dependence on the field strength *F*. Here, $\varkappa =\sqrt{-2E_{0}}$ , with *E*_0_ being the energy of the molecular orbital from which the electron is ionized, and the subscript 00 refers to the dominant ionization channel (Tolstikhin et al., 2011).

The HOMO (1*t*_1_) and HOMO-1 (4*t*_2_) of CF_4_ are both triply degenerate (Figure 3). The Stark interaction with the ionizing field removes the degeneracy. Tunneling ionization occurs from eigenorbitals of the operator –(***μ*** ⋅ ***F***) within each degenerate subspace, where ***μ*** is the electric dipole moment of the considered orbital (Kraus et al., 2015). We denote these eigenorbitals as *ϕ*_A_, *ϕ*_B_, and *ϕ*_C_. The three eigenorbitals are the particular linear combinations of the three degenerate HOMOs shown in Figure 3, which diagonalize the Stark term (***μ*** ⋅ ***F***). The structure factors *G*_00_(*β*, *γ*) incorporating the effect of the dipole for the eigenorbitals are calculated using the integral representation of the WFAT (Dnestryan and Tolstikhin, 2016; Madsen et al., 2017; Dnestryan et al., 2018) implemented by means of the GAMESS package with a polarization consistent basis set at the pc-4 level (Jensen, 2001).

**FIGURE 6:**
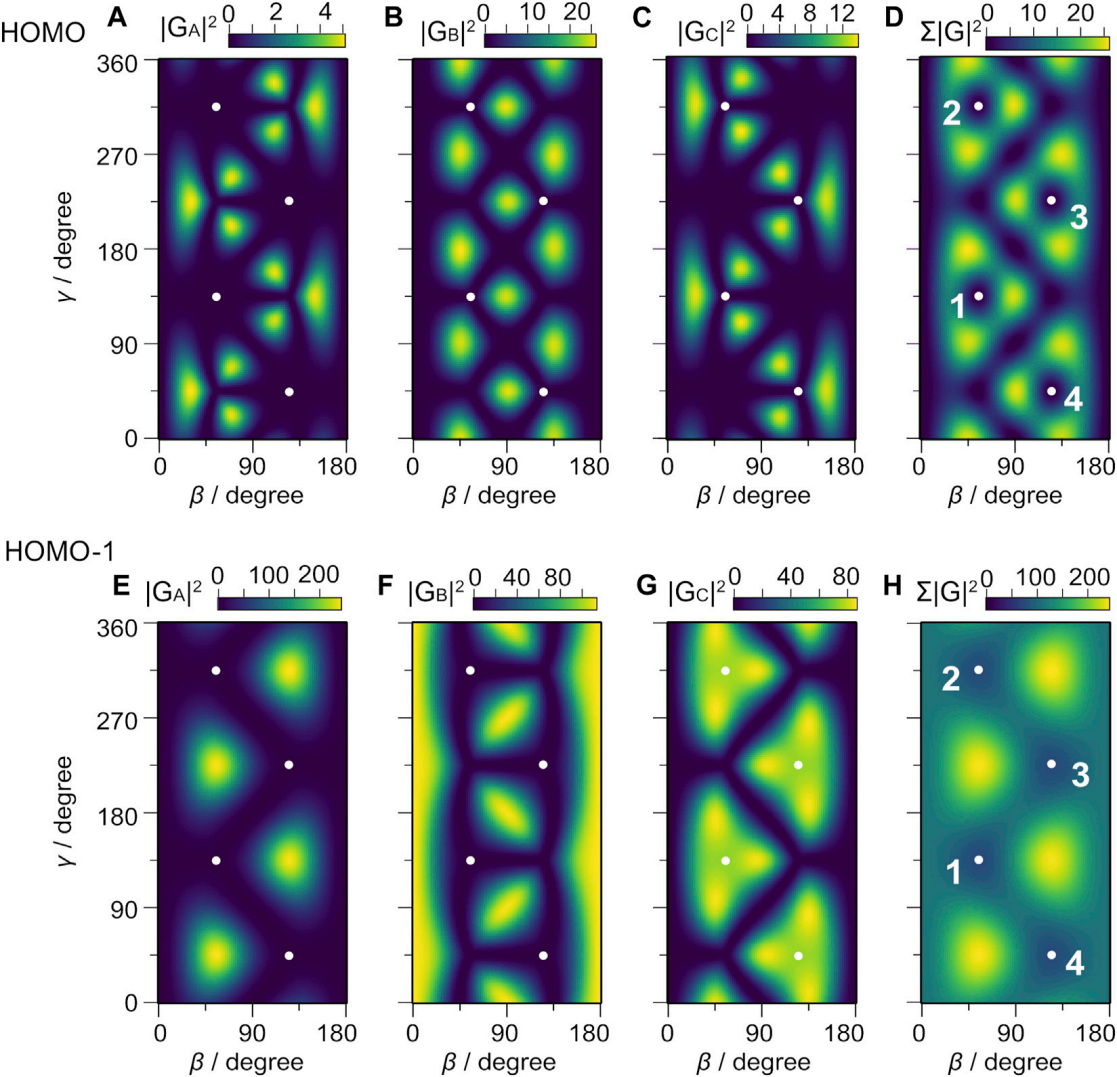
Structure factors of eigenorbitals, **(A)** |*G*_A_|^2^, **(B)** |*G*_B_|^2^, **(C)** |*G*_C_|^2^, and **(D)** the sum, Σ|*G*|^2^ = |*G*_A_|^2^ + |*G*_B_|^2^ + |*G*_C_|^2^ for HOMO, and **(E)** |*G*_A_|^2^, **(F)** |*G*_B_|^2^, **(G)** |*G*_C_|^2^ and **(H) **Σ|*G*|^2^ for HOMO-1. The dots represent the Euler angles (*β*, *γ*) at which one of the C-F axes points to the *Z* direction. The numbers attached to the dots in panels **(D,H)** represent the labels of the respective F atoms in Figure 2. Note the difference in the scaling of the color bars in **(A–H)**.

**FIGURE 7:**
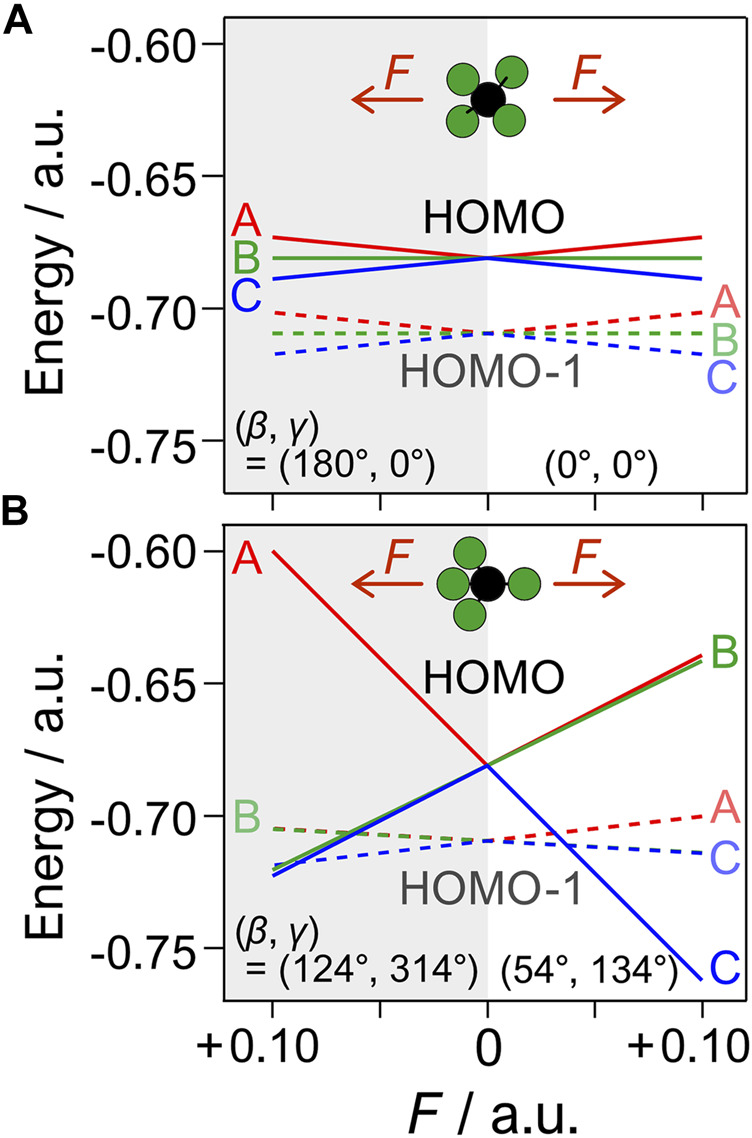
Stark shifted energies of *E*_0,*i*_(***F***) of eigenorbitals, *ϕ*_*i*_ (*i* = A, B, C), of HOMO (solid line) and HOMO-1 (dashed line) as a function of the static field *F* (see Eq. 9) at four different molecular orientations with respect to *F*, defined by the Euler angles (*β*, *γ*) in Figure 2. **(A)** The electric field is parallel to the molecular principal axis (*C*_2_), i.e., (*β*, *γ*) = (0°, 0°) (right) and (180°, 0°) (left). **(B)** The same as **(A)** but for (*β*, *γ*) = (54°,134°) (right) and (124°, 314°) (left), where the electric field is almost parallel to one of the C-F axes.

Figures 6A–C show the squared norms of the structure factors |*G*_00_(*β*, *γ*)|^2^ of the three eigenorbitals, *ϕ*_A_, *ϕ*_B_, and *ϕ*_C_, in the subspace of HOMO (*E*_0_ = –18.66 eV), where the orbitals are labeled with A, B, and C in the ascending order of the dipole, *μ*_A_ < *μ*_B_ < *μ*_C_. The orbital energy in the field to the first order is given as

$$E_{0,i}\left(F\right)=E_{0}-\mu_{i}\cdot F,$$ (9)

where *i* = A,B,C. Figure 7 shows the energy of eigenorbitals calculated using Eq. 9 at four different molecular orientations with respect to *F*. The structure factors for HOMO in Figures 6A–C show that the largest contribution to the tunneling ionization comes from eigenorbital *ϕ*_B_ because the field factor *W*_00_(*F*) is common for *ϕ*_A_, *ϕ*_B_, and *ϕ*_C_ (see Eq. 7). Each orbital has nodes along the C-F axis, which appear as the minima in the respective structure factors. The nodes remain visible in the sum of |*G*_00_(*β*, *γ*)|^2^ in Figure 6D.

The squared norms of the structure factors |*G*_00_(*β*, *γ*)|^2^ of HOMO-1 (*E*_0_ = –19.44 eV) are shown in Figures 6E–H. The eigenorbital *ϕ*_A_ having the highest energy among the three orbitals has the largest contributions to the sum in Figure 6H. Figure 6H shows that the tunneling ionization is enhanced by the electric field pointing from F to C when the three degenerated eigenorbitals are equally populated. Because eigenenergy *E*_0,*A*_ of *ϕ*_A_ at (*β*, *γ*) = (124°, 314°) is slightly smaller than that at (*β*, *γ*) = (54°,134°), the large structure factors for the former orientation indicate that the shape of the molecular orbital is more important in determining the tunneling rate in the present case than the increase in the effective ionization potential by the Stark interaction with the dipole moment.

#### 3.3.2 Fragment Angular Distribution

If the breaking of each of the four C-F bonds after ionization occurs with an equal probability, the angular distribution of the F fragment in the laboratory frame can be expressed as follows (Zare, 1988):

$$P\left(\theta_{s},\phi_{s}\right)=\int_{0}^{2\pi }d\alpha \int_{0}^{\pi }\sin\beta d\beta \int_{0}^{2\pi }d\gamma P_{\text{mol}}\left(\alpha ,\beta ,\gamma \right)f\left(\theta_{m},\phi_{m}\right),$$ (10)

where (*θ*_*s*_, *ϕ*_*s*_) and (*θ*_*m*_, *ϕ*_*m*_) are the spherical angles with respect to the laboratory and molecular frame, respectively, and *f*(*θ*_*m*_, *ϕ*_*m*_) is the angular distribution of the fragment ion in the molecular frame. The orientation distribution of the molecular ion formed in the *ω*-2*ω* laser fields in the laboratory frame may be expressed as

$$P_{\text{mol}}\left(\alpha ,\beta ,\gamma \right)=\frac{1}{8\pi^{2}}\left\{1-\exp\left[-\int_{-\infty }^{+\infty }\Gamma_{s}\left(\alpha ,\beta ,\gamma ,F\left(t\right)\right)dt\right]\right\},$$ (11)

where Γ_*s*_(*α*, *β*, *γ*, *F*(*t*)) represents the tunneling rate in the *ω*-2*ω* laser field *F*(*t*) of Eq. 2 for molecular orientation defined by the Euler angles (*α*, *β*, *γ*) relative to the *Z*-axis of the laboratory frame (see Figure 2). It can be expressed by |*G*_00_(*β*, *γ*)|^2^ and *W*_00_(*F*) as follows:

$$\Gamma_{s}\left(\alpha ,\beta ,\gamma ,F\left(t\right)\right)=\left\{\begin{array}{cc} |G_{00}\left(\beta ,\gamma \right){|}^{2}W_{00}\left(\left|F\left(t\right)\right|\right)\; & \left(F\left(t\right)\geq 0\right)\\ |G_{00}\left(\pi -\beta ,\gamma +\pi \right){|}^{2}W_{00}\left(\left|F\left(t\right)\right|\right)\; & \left(F\left(t\right)<0\right) \end{array}\right..$$ (12)

When the ionization probability is sufficiently smaller than unity, Eq. 11 reduces to

$$P_{\text{mol}}\left(\alpha ,\beta ,\gamma \right)=\frac{1}{8\pi^{2}}\int_{-\infty }^{+\infty }\Gamma_{s}\left(\alpha ,\beta ,\gamma ,F\left(t\right)\right)dt.$$ (13)

The angular distribution *P*_mol_(*α*, *β*, *γ*) can be expanded by the rotation matrices $D_{q^{\prime }q}^{k}\left(R\right)$ as follows:

$$P_{\text{mol}}\left(\alpha ,\beta ,\gamma \right)=\frac{1}{8\pi^{2}}\underset{k,q,q^{\prime }}{\sum }a_{q^{\prime }q}^{k}D_{q^{\prime }q}^{k*}\left(\alpha ,\beta ,\gamma \right).$$ (14)

Here, the coefficients $a_{q^{\prime }q}^{k}$ are given as follows:

$$a_{q^{\prime }q}^{k}=\left(2k+1\right)\int P_{\text{mol}}\left(\alpha ,\beta ,\gamma \right)D_{q^{\prime }q}^{k}\left(R\right)d\Omega .$$ (15)

The angular distribution of the fragment ion can be expressed using the spherical harmonics *Y*_*jm*_(*θ*_*m*_, *ϕ*_*m*_):

$$\begin{array}{ccc} & f\left(\theta_{m},\phi_{m}\right)=\underset{j,m}{\sum }b_{jm}Y_{jm}\left(\theta_{m},\phi_{m}\right), & \end{array}$$ (16)

$$\begin{array}{ccc} & b_{jm}=\int_{0}^{2\pi }d\phi_{m}\int_{0}^{\pi }\sin\theta_{m}d\theta_{m}Y_{jm}^{*}\left(\theta_{m},\phi_{m}\right)f\left(\theta_{m},\phi_{m}\right). & \end{array}$$ (17)

Thus, we have

$$P\left(\theta_{s},\phi_{s}\right)=\underset{k,q,q^{\prime }}{\sum }\frac{a_{q^{\prime }q}^{k}b_{kq}}{2k+1}Y_{kq^{\prime }}\left(\theta_{s},\phi_{s}\right).$$ (18)

Under the axial recoil approximation, the angle distribution *f*(*θ*_*m*_, *ϕ*_*m*_) may be expressed as follows:

$$f\left(\theta_{m},\phi_{m}\right)=\frac{1}{\sin\theta_{m}}\delta \left(\theta_{m}-\theta_{m}^{0}\right)\delta \left(\phi_{m}-\phi_{m}^{0}\right),$$ (19)

with $\left(\theta_{m}^{0},\phi_{m}^{0}\right)$ = (54.7°, 45°) for CF_4_ in *T*_d_ symmetry. By substituting to Eq. 17, we have

$$b_{jm}=Y_{jm}^{*}\left(\theta_{m}^{0},\phi_{m}^{0}\right)={\left(-1\right)}^{m}Y_{j-m}\left(\theta_{m}^{0},\phi_{m}^{0}\right),$$ (20)

from which we obtain an expression for the fragment angular distribution as follows:

$$\begin{array}{ccc} & P\left(\theta_{s},\phi_{s}\right)=P\left(\theta_{s}\right)=\frac{1}{\sqrt{4\pi }}\underset{k}{\sum }c_{k}P_{k}\left(\cos\theta_{s}\right), & \end{array}$$ (21)

$$\begin{array}{ccc} & c_{k}=\frac{1}{\sqrt{2k+1}}\underset{q}{\sum }a_{0q}^{k}Y_{kq}^{*}\left(\theta_{m}^{0},\phi_{m}^{0}\right). & \end{array}$$ (22)

**FIGURE 8:**
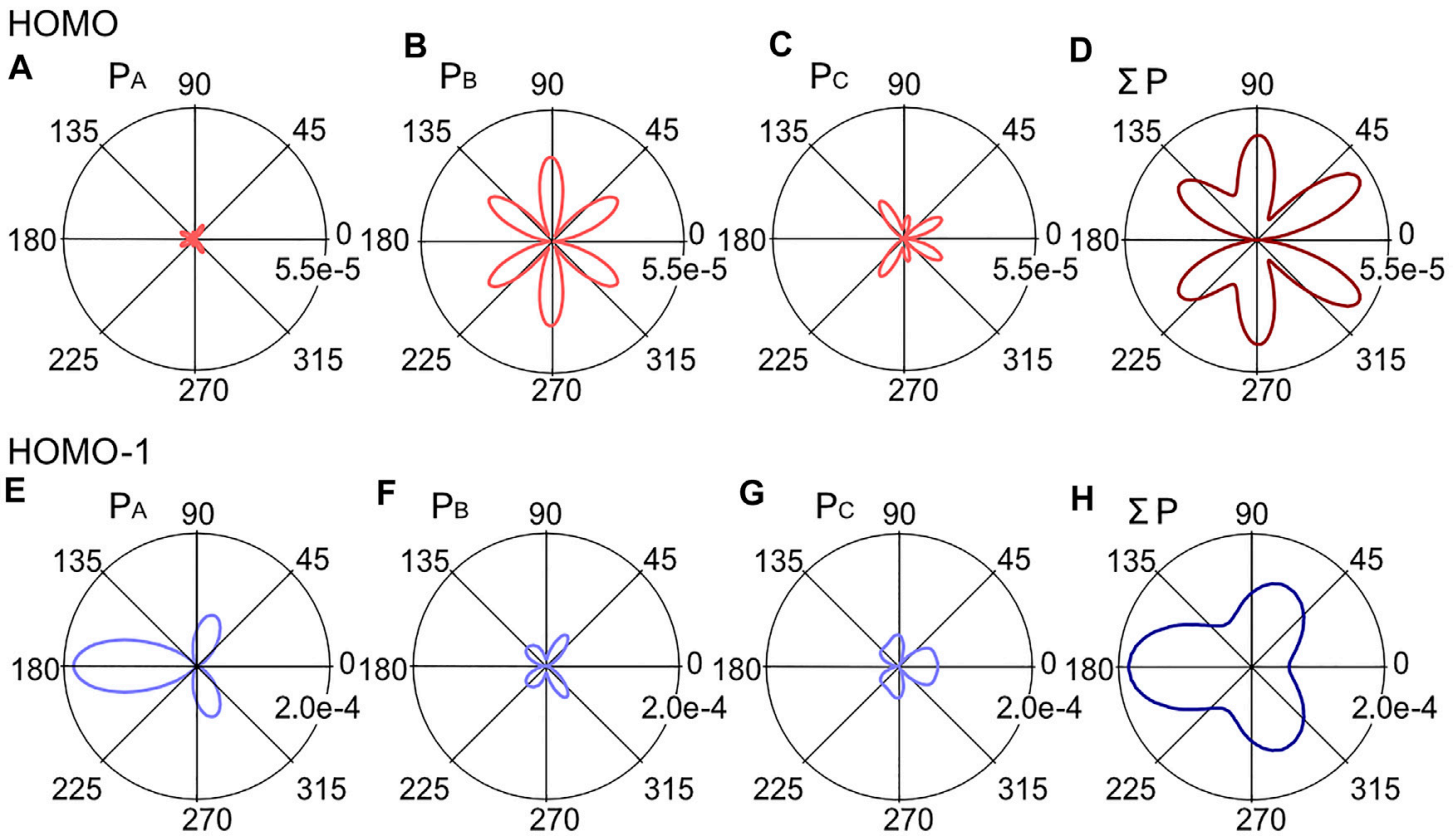
Angular distributions *P*(*θ*_*s*_) calculated for the eigenorbitals, *ϕ*_A_, *ϕ*_B_, and *ϕ*_C_, in the *ω*-2*ω* laser field with the relative phase *ϕ* = 0, **(A)** *P*_A_(*θ*_*s*_), **(B)** *P*_B_(*θ*_*s*_), **(C)** *P*_C_(*θ*_*s*_), and **(D)** the sum Σ*P*(*θ*_*s*_) for HOMO and **(E)** *P*_A_(*θ*_*s*_), **(F)** *P*_B_(*θ*_*s*_), **(G)** *P*_C_(*θ*_*s*_) and **(H)** Σ*P*(*θ*_*s*_), for HOMO-1. Note the difference in the scaling of the polar plots for HOMO and HOMO-1.

Figure 8 shows the fragment angular distributions obtained for the relative phase *ϕ* = 0 of the *ω* - 2*ω* pulse (*I*_*ω*+2*ω*_ = 1.4 × 10^14^ W/cm^2^ and *I*_2*ω*_/*I*_*ω*_ = 0.23). The calculated fragment yields for HOMO-1 is larger than that of HOMO under the present experimental conditions (*F*_*ω*_ = 0.057 a.u. and *F*_2*ω*_ = 0.027 a.u.).

This is attributed to the large structure factor |*G*_00_|^2^ for HOMO-1 (Figure 6H), which is about 10 times larger than |*G*_00_|^2^ for HOMO (Figure 6D), because of the small difference between the ionization potentials of these orbitals (∼1 eV) giving rise to the relatively small field factor ratio of *W*_00_(1*t*_1_)/*W*_00_(4*t*_2_) ∼ 3. The angular distribution calculated for each HOMO exhibits characteristic structures associated with the nodes of the molecular orbitals. The total fragment distribution carries the nodal pattern with a larger ionization probability on the larger amplitude side of the *ω*-2*ω* laser fields. In contrast, the angular distribution of HOMO-1 is more directional along the laser polarization direction, consistent with the fragment ion image and the KER spectra in Figures 5A,B, where the ionization from HOMO-1 contributes more to the parallel component than to the perpendicular one.

#### 3.3.3 Asymmetry Parameter

The yields of the F fragment in a finite acceptance angle *θ*_0_ around 0° and 180° can be expressed as follows:

$$\begin{array}{ccc} & Y_{+}^{\theta_{0}}\left(\phi \right)=2\pi \int_{0}^{\theta_{0}}P\left(\theta_{s}\right)\sin\theta_{s}d\theta_{s}, & \end{array}$$ (23)

$$\begin{array}{ccc} & Y_{-}^{\theta_{0}}\left(\phi \right)=2\pi \int_{\pi -\theta_{0}}^{\pi }P\left(\theta_{s}\right)\sin\theta_{s}d\theta_{s}. & \end{array}$$ (24)

**FIGURE 9:**
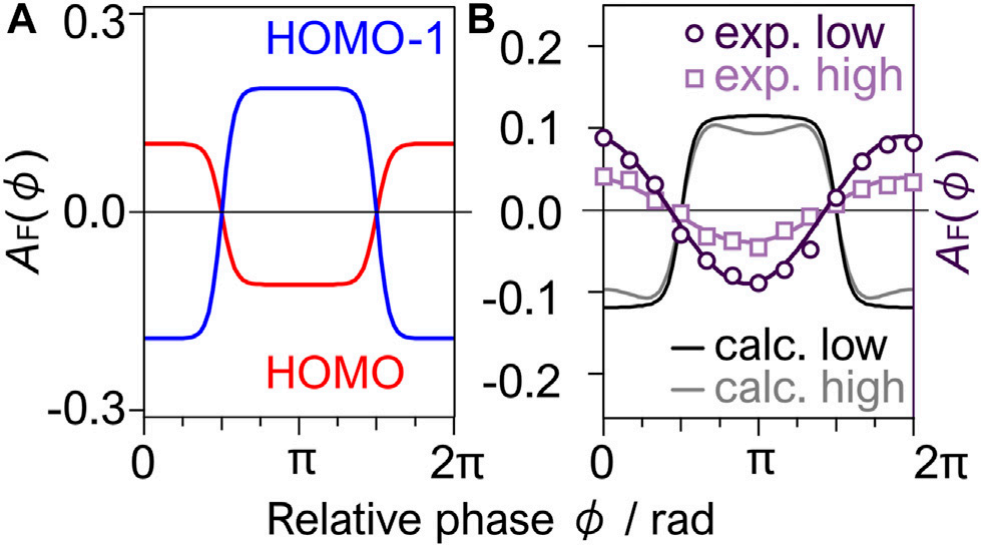
**(A)** Phase dependence of the asymmetry parameter *A*(*ϕ*) calculated for HOMO and HOMO-1 with the acceptance angle of *θ*_0_ = 45°. **(B)** Total asymmetry parameter compared with the experimental parameter for F fragment, *A*_F_(*ϕ*), calculated at *I*_*ω*+2*ω*_ = 1.4 × 10^14^ W/cm^2^ (solid) and 3.0 × 10^14^ W/cm^2^ (gray), in comparison with the corresponding experimental results (circle and square, respectively).

The asymmetry parameters defined by Eq. 5 are calculated using Eqs 23, 24, where *θ*_0_ = 45° compared with the experimental results. The asymmetry parameter *A*_F_(*ϕ*) thus obtained for HOMO shows a clear dependence on the relative phase *ϕ* between the *ω* and 2*ω* laser fields. The asymmetry parameter for HOMO (Figure 9A) is positive at *ϕ* = 0, showing that tunneling ionization is more efficient when the larger amplitude side of the laser fields points from C to F. In contrast, the parameter for HOMO-1 exhibits the opposite dependence with negative values at *ϕ* = 0. The difference originates essentially from the shape of the eigenorbitals dominating the tunneling ionization of the respective MOs.

Figure 9B plots the experimental asymmetry parameter *A*_F_(*ϕ*) for the counterpart fragment F produced by the dissociative ionization (Eq. 3), which is obtained from the asymmetry parameter for ${CF}_{3}^{+}$ by $A_{F}\left(\phi \right)=-A_{{\text{CF}}_{3}^{+}}\left(\phi \right)$ . It is compared with the corresponding asymmetry parameter calculated with the contributions from the two orbitals, where the angular distribution is given as *P*(*θ*_*s*_) = *P*_HOMO_(*θ*_*s*_) + *P*_HOMO-1_(*θ*_*s*_). The obtained amplitude of *A*_0_ = 0.1 is slightly larger than the experimental results. The small experimental amplitude might be attributed to the contribution from HOMO-2 (1*e*), located ∼1.4 eV below the HOMO-1. The B^2^E state of ${CF}_{4}^{+}$ produced by the tunneling ionization from 1*e* has a lifetime of 10^–10^–10^–12 ^s (Maier and Thommen, 1980). This lifetime is longer than or comparable with the molecular rotational periods and could form an isotropic fragment distribution, which reduces the asymmetry of the fragmentation. Figure 9B plots the asymmetry parameter *A*_F_(*ϕ*) obtained at a higher field intensity 3.0 × 10^14^ W/cm^2^ with a similar intensity ratio of *I*_2*ω*_/*I*_*ω*_ = 0.25. The increase in the field intensity resulted in a small amplitude *A*_0_ ∼ 0.04, while the amplitude of the calculated results remained essentially the same. Because the relative contribution from the B^2^E state is expected to increase by an increase in the field intensity, the experimental results support the involvement of the B^2^E state in the dissociative ionization.

Interestingly, the calculated asymmetry parameter in Figure 9B has an opposite phase dependence to the experimental results, showing that the dissociative tunneling ionization of CF_4_ in the *ω*-2*ω* laser fields cannot be explained by the angular distribution of the tunneling ionization from the HOMO and HOMO-1 alone, although the F (or ${CF}_{3}^{+}$ ) fragments are promptly ejected on the repulsive potentials of the X^2^T_1_ and A^2^T_2_ states after the tunneling ionization (Figure 4). The present experimental results show a marked contrast to those obtained by recent studies on the dissociative ionization of CF_4_ in circularly polarized laser fields (35 fs, 0.8 × 10^14^ W/cm^2^, 1,035 nm) (Fujise et al., 2022). The recoil-frame photoelectron angular distribution (RFPAD) showed that the dissociative tunneling ionization occurs more efficiently when the electric field points from F to C than the opposite, which is consistent with the prediction by WFAT for the tunneling ionization (see also Figure 8).

Previous studies on spatially oriented OCS showed that the tunneling ionization yields exhibit different angular dependence in linearly polarized and circularly polarized laser fields (Holmegaard et al., 2010; Hansen et al., 2012) as in the present case, where the tunneling ionization is enhanced at different directions of the applied electric fields in the molecular frame. For circularly polarized fields, a significant enhancement of tunneling ionization was observed when the electric fields were applied from C to S along the molecular axis, while the linearly polarized fields favor the tunneling ionization from the direction perpendicular to the axis. The discrepancy was discussed in terms of electron rescattering and the involvement of electronic excitation (Hansen et al., 2012), as well as orbital modification (Murray et al., 2010) and multielectron effects (Majety and Scrinzi, 2015) in the ionization process. These effects can, in principle, be involved in the present case of CF_4_ to explain the deviation between the experimental and theoretical results in Figure 9B. Furthermore, Figure 7 suggests that the energy shifts of eigenorbitals formed by the Stark interaction becomes large enough to induce mixing between HOMO and HOMO-1, for example, at a field intensity *F* ≥ 0.06 a.u. in the molecular orientation in Figure 7B. This would result in additional polarization (field-induced deformation) of the ionizing orbitals, which affects the ionization rate (Matsui et al., 2021) but is not considered in the calculation of the structure factors in Figure 6.

Because the directional ejection of the fragments involves both ionization and fragmentation, post-ionization interaction with the laser fields (Endo et al., 2019; Endo et al., 2022) is another important factor to consider. The post-ionization interaction in *ω*-2*ω* laser fields has been extensively studied with $H_{2}^{+}$ (Ray et al., 2009; Wanie et al., 2015). The dissociative ionization shows a clear dependence on the relative phase *ϕ*. The H^+^ ejection direction is determined by the quantum interference between the pathways associated with excitation and deexcitation between the 1s*σ*_*g*_ and 2p*σ*_*u*_ states of $H_{2}^{+}$ by absorption or emission of *ω* and 2*ω* photons. This results in the spatial asymmetry of H^+^ ejection dependent on both phase *ϕ* and KER. The quantum interference effect can also manifest itself in circularly polarized laser fields when the tunneling electron is detected in coincidence with H^+^ (Wu et al., 2013). It appears as the distortion of the molecular-frame photoelectron angular distribution (MFPAD). As for CF_4_, the RFPADs recorded for the dissociative ionization in Eq. 3 in circularly polarized fields exhibited clear dependences on both the helicity of circularly polarized laser fields and the KER ^1^. The observed results are interpreted in terms of the laser-induced coupling between the electronic states, depending on the phase of the rotating electric fields in the molecular frame. The coupling between the ground state X^2^T_1_ and the excited state A^2^T_2_ through non-adiabatic population transfer in the alternating laser electric fields was suggested as a possible dynamics contributing to the helicity dependence. In the present case of the two-color laser fields consisting of 800 and 400 nm for *ω* and 2*ω*, the energy differences between the states and the A^2^T_2_ and B^2^E states are close to the photon energy of *hν* = 1.5 and 3.1 eV of the present *ω* and 2*ω* fields (see Figure 4), which further facilitates such coupling to modify the asymmetry of the fragmentation through quantum interferences.

## 4 Summary

In the present study, we investigated the directional fragment ejection of CF_4_ in dissociative ionization, CF_4_ → ${CF}_{3}^{+}$ + F + e^−^, in linearly polarized *ω*-2*ω* ultrashort intense laser fields (1.4 × 10^14^ W/cm^2^, 800 and 400 nm) by three-dimensional ion momentum imaging. The ${CF}_{3}^{+}$ fragment distribution exhibited a clear dependence on the relative phase *ϕ* between the *ω* and 2*ω* laser fields, showing that the ${CF}_{3}^{+}$ ions tend to be ejected to smaller electric field sides of the two-color laser fields. The observed results indicated that the asymmetric ejection of the ${CF}_{3}^{+}$ ion or the F fragment can be manipulated by the relative phase of the *ω*-2*ω* intense laser fields. To understand the mechanism of the directional fragment ejection, the tunneling ionization rates were calculated by the weak-field asymptotic theory (WFAT) incorporating the Stark interaction in the triply degenerated orbitals of HOMO and HOMO-1. It was shown that the contributions from the HOMO-1 (4*t*_2_) are even larger than those from HOMO (1*t*_1_). The inverted order is attributed to the large structure factor of HOMO-1, which is governed essentially by the shape of the MO. The observed momentum distribution of ${CF}_{3}^{+}$ and the KER spectrum supported that both the X^1^T_1_ and A^2^T_2_ states contribute to the dissociative ionization of CF_4_ in the *ω*-2*ω* intense laser fields.

In contrast, WFAT showed that the ionization yield sum becomes larger when the electric field points from F to C along the one of the C-F axis to predict a phase-dependent asymmetry parameter *A*(*ϕ*) being *π* out-of-phase to the experimental one. The difference between experimental and theoretical results could be attributed to additional distortion of molecular orbitals by mixing between HOMO and HOMO-1, as well as to the other processes proposed in the previous studies. The post-ionization process is another possible source of different phase dependence. The direct coupling between the electronic states of ${CF}_{4}^{+}$ by non-adiabatic transitions between the orbitals would cause constructive and destructive interference of the dissociating nuclear wavepackets to make the four C-F bonds inequivalent in dissociation. The present study demonstrated the feasibility of applying strong-field coherent control of directional fragment ejection to a symmetric polyatomic molecule in *T*_d_ symmetry. Several factors need to be considered to fully understand the selective breaking of C-F bonds in the dissociative tunneling ionization, even though ultrafast dissociation occurs on the repulsive potential surfaces after the ionization.

## Data Availability

The original contributions presented in the study are included in the article/Supplementary Material. Further inquiries can be directed to the corresponding author.
